# A Common Molecular Signature Indicates the Pre-Meristematic State of Plant Calli

**DOI:** 10.3390/ijms241713122

**Published:** 2023-08-23

**Authors:** Attila Fehér

**Affiliations:** 1 Institute of Plant Biology, Biological Research Centre, 62 Temesvári Körút, 6726 Szeged, Hungary; feher.attila@brc.hu or feher.attila.sandor@szte.hu; 2 Department of Plant Biology, University of Szeged, 52 Közép Fasor, 6726 Szeged, Hungary

**Keywords:** callus, gene expression, organ primordium, meristem, transcription factor, auxin, cytokinin, plant regeneration

## Abstract

In response to different degrees of mechanical injury, certain plant cells re-enter the division cycle to provide cells for tissue replenishment, tissue rejoining, de novo organ formation, and/or wound healing. The intermediate tissue formed by the dividing cells is called a callus. Callus formation can also be induced artificially in vitro by wounding and/or hormone (auxin and cytokinin) treatments. The callus tissue can be maintained in culture, providing starting material for de novo organ or embryo regeneration and thus serving as the basis for many plant biotechnology applications. Due to the biotechnological importance of callus cultures and the scientific interest in the developmental flexibility of somatic plant cells, the initial molecular steps of callus formation have been studied in detail. It was revealed that callus initiation can follow various ways, depending on the organ from which it develops and the inducer, but they converge on a seemingly identical tissue. It is not known, however, if callus is indeed a special tissue with a defined gene expression signature, whether it is a malformed meristem, or a mass of so-called “undifferentiated” cells, as is mostly believed. In this paper, I review the various mechanisms of plant regeneration that may converge on callus initiation. I discuss the role of plant hormones in the detour of callus formation from normal development. Finally, I compare various Arabidopsis gene expression datasets obtained a few days, two weeks, or several years after callus induction and identify 21 genes, including genes of key transcription factors controlling cell division and differentiation in meristematic regions, which were upregulated in all investigated callus samples. I summarize the information available on all 21 genes that point to the pre-meristematic nature of callus tissues underlying their wide regeneration potential.

## 1. Introduction

Higher plants (referred to only as “plants” hereinafter), as sessile organisms, are repeatedly exposed to abiotic factors (e.g., wind, fire) and biotic agents (e.g., herbivores) that damage their structural and functional integrity. Plants can cure smaller damages via tissue repair, during which they reconnect severed organs, such as stems and petioles [1,2]. However, the damage can result in the loss of large parts of the plant involving one or several organs. Therefore, plants have evolved so that they do not have irreplaceable individual organs, such as the brain or the heart of animals, that would cause them to die if lost. Instead, plants have a modular design that ensures their survival even if several of their numerous identical organizational units, called “phytomers” [3], are lost. Phytomers are constantly produced by the apical meristems underlying plant growth and replacing old, non-functional, or lost organs with new ones. The phytomers are not only structural but also fully functional developmental units. They themselves have post-embryonically formed meristems, which, besides allowing branching, can replace the apical meristem if lost [4,5]. However, the regeneration ability of plants goes far beyond activating preexisting dormant meristems. New meristems can de novo form from differentiated tissues of adult plants. For example, when the tip of a plant shoot or root is partially or entirely removed, the cells at the wound site undergo a process of reprogramming, restoring the apex that contains a new apical meristem [1,2,6,7,8,9,10]. Meristems can even develop at places where no meristem existed before, leading to adventitious root, shoot, or even embryo formation [11]. The regeneration ability of higher plants is so extreme that even a single cell can be regenerated into a whole plant, although only under artificial in vitro conditions [12,13,14].

The extreme regeneration ability of plants is widely explored for biotechnological applications, including cloning and mass propagation of selected individuals, production of virus-free plants, grafting, and genetic modifications at the cell level (mutagenesis, cell hybridization, genetic transformation, genome editing, etc.) followed by the regeneration of genetically modified whole plants [15,16,17]. Due to the wide impact of this technology on plant breeding and biotechnology, in vitro regeneration systems have been elaborated for countless plant species. Nevertheless, despite the considerable recent progress in the understanding of the molecular background of plant regeneration [18,19,20], there are still significant gaps in our knowledge, preventing the genotype-independent general application of the technology [21].

Many in vitro plant regeneration systems include the intermediate formation of so-called callus tissue. Besides serving as an intermediary phase in plant regeneration, callus cells can be cultured as cell suspensions in various bioreactors to produce useful metabolites [22]. Although plant callus cultures are central in many plant biotechnology applications, the definition of a plant callus tissue is a question of debate. The reason behind it is that calli can form in various ways and exhibit various characteristics, depending on the plant species, genotype, source tissue, induction, and culture conditions, etc. [21,23]. The general view is that a plant callus is an unstructured non-specialized tissue formed by dividing parenchymatic cells. Furthermore, the callus is often, and wrongly, considered a “dedifferentiated” cell mass [21]. In this review, I aim to update the knowledge about callus formation and discuss whether callus cells have characteristic gene expression markers that define them as a special tissue.

## 2. Callus Formation

Plant callus forms naturally as a wound-healing tissue [24,25] or as a plant tumor in response to pathogens or genetic disorders [26]. In vitro, callus formation is achieved via the application of plant hormones, with or without wounding (for review [23,27]. The appropriate ratio of auxin and cytokinin is considered the most potent inducer of it [28,29]. A complex network of transcription factors (TFs) is implicated in callus initiation (for review [23]). In agreement, callus formation also occurs in plants due to the ectopic or missing expression of key genes involved in developmental regulation (for review [23]). Environmental cues, including light, temperature, and the presence of pathogens, also influence the formation of calli [1]. In agreement with the various explants and conditions used for callus initiation and culture, plant calli are not uniform, neither morphologically nor in their developmental potential. They can vary in color (white, yellow, green, brown), their compact or friable structure, their dependence on plant hormones for cell division, and their regeneration potential (non-regenerating, rooty, shooty, or embryogenic) [26]. Based on the diversity of callus tissues, it is not surprising that the initial steps of their formation can also be quite different. Below I overview the molecular control of wound and auxin-induced callus formation. Details about tumor formation in plants due to genetic and/or environmental perturbations of plant development can be found in the recent review of Dodueva et al. [26] and will not be discussed here.

### 2.1. Tissue Regeneration and Wound Healing

As highlighted above, one of the efficient inducers of callus development is wounding. Although many steps of wound healing in plants are still obscure, current experimental evidence provides considerable insight into its molecular mechanism. Depending on the site and extent of the wound, callus formation might have different roles. It can serve to facilitate the physical closure of the wound, promote the connection between the wound surfaces, and provide cells that can differentiate into various cell types to replenish lost tissues [1]. Single-cell ablation or excision at the meristematic region induces cell/tissue regeneration without visible callus formation. These cases are characterized by limited division of proliferation competent cells directly followed by cell fate transitions replenishing lost cells/tissues in an organized way. Nevertheless, the well-organized cell/tissue replenishment and the less-organized callus formation might share the first steps leading to the activation of cell division. Therefore, I briefly overview both processes. For more comprehensive reviews on plant regeneration, see [1,2,10,20,30,31,32,33].

#### 2.1.1. In the Root

Injury within the root meristem induces jasmonic acid signaling at the wound site leading to the activation of the genes coding for the ETHYLENE RESPONSE FACTOR 109 (ERF109) and, subsequently, the ERF115 TFs [33,34,35]. ERF115 is considered a significant regulator in the activation of regenerative cell divisions in competent root cells [36,37]. In unwounded roots, ERF115 and ERF114 increase auxin sensitivity in response to mechanical cues, thus facilitating the formation of lateral roots (LRs) [38]. If a cell is dying in the root meristem, it is losing turgor and collapsing, generating a mechanical signal that results in local auxin accumulation as well as auxin sensitivity in wound-adjacent cells [39,40]. The mechanical and auxin signals then cooperatively and spatially activate the ERF115 factor in endodermal and stele cells next to the wound. ERF115 promotes the expression of the AUXIN RESPONSE FACTOR 5 (ARF5 or MONOPTEROS), thus further sensitizing the cells for wound-induced auxin accumulation and granting them stem cell activity [37]. In agreement, ERF115-deficient plants are unable to localize auxin biosynthesis at the site of root injury [40]. Thus ERF115 acts synergistically with auxin during the regeneration of root stem cells [37,40]. ERF115 controls root tip regeneration in complex with the PHYTOCHOME A SIGNAL TRANSDUCTION 1 (PAT1) GRAS-type TF [36]. One of the downstream targets is the gene coding for the AP2/ERF-type TF WOUND-INDUCED DEDIFFERENTIATION 1 (WIND1), a member of the WIND1-4 family of regulators involved in cell fate transitions including callus formation [23,36,41,42,43]. However, neither *PAT1* nor *WIND1* exhibited altered expression, and *WIND1* expression did not interfere with restorative cell divisions if only a single cell was removed by laser ablation [9]. Thus, the extent of the wound may influence regeneration pathways downstream of ERF115.

In the Arabidopsis root, cell/tissue regeneration competence decreases with the distance from the root meristem dependent on the gene expression dosage of the PLETHORA 2 (PLT2) TF [9,44]. PLT2 is responsible for the maintenance of the root meristem, and it controls the sequential fate transitions from stem cells to transit amplifying cells and then to the differentiating ones [45]. In the unwounded root meristem, the expression of *ERF115* is confined only to the quiescent center (QC). However, experimental data support the view that in response to increasing levels of reactive oxygen species (ROS), the expression of *PLT2* is downregulated, and that of *ERF115* expands to the whole root meristem [46]. Thus wound-induced ROS and jasmonic acid signaling might converge on the expression of *ERF115* in a PLT2-dependent way mediating meristem-specific regenerative cell divisions. If the wound affects the root differentiation zone, which has low PLT2 expression and thus low regeneration competence, ethylene rather than jasmonic acid is the major stress hormone produced [2]. Unlike in the meristematic region, there is no local persistent auxin accumulation nor subsequent cell activation in response to wounding in this region [40]. Elevated *PLT2* expression [44], as well as exogenous auxin, can improve the tissue regeneration competence of non-meristematic root zones [40]. These observations demonstrate the importance of local auxin synthesis, which is spatially controlled, among other things, by PLT2 and ERF115, in the regenerative response of root cells.

#### 2.1.2. In the Shoot

Shoot tip regeneration is obviously a cytokinin-driven process, although the signaling steps of it have not yet been revealed [47]. Whether the ERF115/ERF109-WIND1 module is involved in the shoot tip regeneration pathway, such as in the root tip regeneration pathway, is an open question. WIND1 is considered to participate in the activation of cytokinin signaling in response to the injury of the hypocotyl [23,27,42,43]. Wound-induced callus formation on hypocotyls is associated with and requires the increased expression of genes encoding cytokinin biosynthetic enzymes [43]. In agreement, induction of WIND1 expression in Arabidopsis seedlings elevated the expression of cytokinin biosynthesis genes, *LONELY GUY 1* (*LOG1*), and *LOG4* within six hours [48]. The locally activated cytokinin synthesis and WIND1-dependent pathways converge on the activation of cytokinin signaling, including the type-B ARABIDOPSIS RESPONSE REGULATOR 1 (ARR1) and ARR12 proteins and the cell cycle activator CYCLIN D3;1 (CYCD3;1) [41,43]. In addition, a set of AP2/ERF TFs, PLT3, PLT5, and PLT7, act downstream of wound-induced cytokinin, and the *plt357* triple mutant is defective in callus formation [43]. Although the direct link of the PLT3, PLT5, and PLT7-coding genes to cytokinin signaling has not been proven, *PLT5* expression was shown to be induced by WIND1 [48].

Experiments also demonstrated the requirement of *ERF115*/*PAT1* and *WIND1* function for efficient callus formation at the wounded site of the Arabidopsis hypocotyl [43]. Furthermore, induced *WIND1* expression in whole seedlings elevated the expression levels of *ERF115* [48]. Wounding of leaf tissues also enhanced *ERF115* expression within hours [48]. These observations support the view that the ERF115/PAT-WIND1 transcription regulatory module might play a central role in wound-induced tissue regeneration external to the root, too. WIND1, likely in parallel with cytokinin, has been demonstrated to transcriptionally upregulate further regeneration factors, including the ENHANCER OF SHOOT REGENERATION 1 (ESR1), RELATED TO AP2-LIKE (RAP2.6L), ESR2, PLT5, ANAC071, LBD18, WUSCHEL (WUS), WUSCHEL-RELATED HOMEOBOX 13 (WOX13), WOX5, LEAFY COTYLEDON 2 (LEC2) and OBF BINDING PROTEIN 4 (OBP4) to promote callus formation as well as tissue joining and repair [30,48,49].

It is of note; that small stem incisions, not completely preventing the basipetal auxin flow in the shoot axis, do not induce WIND1 expression [30]. Nevertheless, even in these cases, the combined action of auxin, ethylene, and jasmonic acid results in the activation of some of the above regeneration factors. The asymmetric expression of *RAP2.6L* at the bottom cut and the NAC-type TF *ANAC071* at the top cut was found to be required to govern the tissue reunion [50].

WIND1 is also one of the critical factors in the regulation of vascular reconnection following grafting [48]. Despite its action being cytokinin-dependent, the processes it regulates are also heavily influenced by sugars [51,52] and auxins [30,50,52,53,54]. In agreement, ectopic *WIND1* expression was shown to upregulate the auxin synthesis gene *YUCCA4* (*YUC4*) and the auxin transport gene *PINFORMED 1* (*PIN1*) in seedlings [48]. Auxin and sugars were shown to accumulate asymmetrically at the top and bottom of the cut [52]. Callus formation efficiency at the cut sites is also asymmetric such as the expression of the partly WIND1-regulated *WOX13* and *RAP2.6L* genes [50,54]. *WOX13* expression is remarkably high in the vicinity of the cut site of the top part of the shoot, which is associated with increased auxin level and callus formation there; conversely, *RAP2.6L* is strongly expressed at the wound of the bottom part, which is in line with the reduction of auxin concentration there [50,54]. It was shown that WOX13 is required for efficient wound-induced callus formation [53,54]. Wounding (WIND1) and auxin independently activate the expression of *WOX13* in a reinforcing way [54]. WOX13 was found to upregulate the genes of the WIND-family factors WIND2 and WIND3 [53]. Thus, a gene regulatory circuit forms that robustly facilitates cellular reprogramming [53,54]. Since WOX13 is required for and WIND1 promotes the adhesion of organs during grafting, the same molecular mechanisms might be involved in the callus and graft formation processes [48,53]. In leaves, the wound also activates the gene coding for the SET-domain chromatin modifier ASH1-RELATED 3 (ASHR3) [55]. Upon wounding, this protein bestows histone H3 lysine-36 methylation marks to the promoters of the *ARR1*, *PLT3*, and *WIND3* genes, thereby promoting their expression [55]. The *ashr3* mutant is defective in wound-induced callus formation but increases the rate of de novo AR formation of leaf explants [55]. Cutting of the leaf base can anyway result in de novo root formation at a high rate [56]. During this age-dependent [57] and spatially restricted [56] organ regeneration process, temporal accumulation of jasmonic acid induces the expression of ERF109, which enhances the local synthesis of auxin via its precursor tryptophan [34]. Interestingly, auxin biosynthesis in the distal part of the detached leaf, away from the cut site, and its transport to regeneration-competent cells is also an inherent part of the process [52,58].

### 2.2. Exogenous Hormone-Induced Callus Formation

As outlined above, plants can respond to injury by tissue reunion, organ joining, wound healing, cell replenishment, or organ regeneration. All these responses are controlled by hormonal interactions and include the temporal reactivation of cell division in parallel or in advance of cell fate transition. Similar processes can also be artificially provoked underlying the technology of in vitro plant cell and tissue culture. Most in vitro plant cell/tissue cultures include wounding due to the use of explants. Since these explants are liberated from the long-range endogenous signals that control plant regeneration, the appropriate ratio of exogenous plant hormones must be applied to initiate and/or maintain cell divisions as well as to trigger subsequent cell differentiation. Auxins and cytokinins are the key hormones determining cell division and differentiation responses in vitro, such as *in planta*. Although organ regeneration might be achieved in vitro without visible callus formation, in most instances, a callus culture is first established, followed by the induction of organ regeneration or embryogenesis [27,59]. Experiments conducted by Skoog and Miller in 1957 [28] have already demonstrated that the ratio of cytokinin to auxin determines the morphogenetic pathway of in vitro cultured plant tissues. High ratios of cytokinin to auxin result in shoot regeneration, whereas low ratios favor root regeneration. Intermediate ratios, however, result in the unorganized growth of a cell mass, referred to as in vitro callus. Since in the callus-inducing media (CIM), auxin is the prevalent hormone supported by cytokinin [60], in vitro callus induction is generally considered an auxin response. Indeed, callus formation can be achieved without exogenous cytokinins [61], but a certain level of endogenous cytokinin is required even in these cases to maintain cell division activity [62,63]. “Nevertheless, the brassinosteroid hormone could replace cytokinin in the induction of the CycD3 cyclin gene to promote Arabidopsis callus formation [64]. Furthermore, exogenous application of hormones like abscisic acid or ethylene might also promote callus formation, likely via altering the endogenous auxin/cytokinin ratio in the explant [65]”.

During the last fifteen years, considerable progress has been achieved in explaining the auxin-mediated initiation of callus tissues from various explants, primarily using Arabidopsis as a model (for reviews [21,23]). Surprisingly, histological observations, sing root meristem-specific fluorescent protein markers, and Arabidopsis mutants defective in the lateral root (LR) development revealed that auxin-induced callus formation follows the LR development pathway [61,66]. CIM-induced calli originate from pericycle cells of root explants exactly at the sites of LR initiation [61,66], and the tissue layers of the emerging calli display histological characteristics that are reminiscent of the root meristem, including the organized spatial expression of root meristem regulators such as WOX5 [66,67]. Even more surprisingly, placing aerial explants on CIM activates the same LR formation-related pathway in vasculature-associated cell layers [66]. Mutants defective in auxin-mediated LR formation are also defective in callus initiation, supporting the overlap in the signaling steps that include the degradation of the auxin signaling repressor IAA14 and the subsequent activation of the auxin response factors (ARFs) ARF7 and ARF19, which switch on the transcription of the genes coding for a subset of LATERAL ORGAN BOUNDARY (LBD) TFs including LBD16, LBD17, LBD18, and LBD29 [23,27,41,68,69,70,71]. LBD factors are central players in LR formation as well as in callus formation [70,71]. These factors, among others, promote cell proliferation (through transcriptional activation of the cell cycle regulator E2 PROMOTER BINDING FACTOR an (E2Fa) [72]) and the remodeling of the cell walls [73,74,75] to facilitate the emergence of either the root primordium or the callus. LBDs are also implicated in adventitious root (AR) initiation [76]. During AR development, WOX11/12 activates *LBD16* and *WOX5*, which govern the de novo formation of root primordia [58,76]. Adventitious rooting can serve as an alternative pathway for callus initiation on CIM [67,76,77,78]. Thus, callus formation on CIM is a detour from the root primordium formation, irrespective of the very first steps leading to either LR or AR development.

One of the intriguing discoveries of these studies was that CIM-induced callus originates from specific pre-existing competent cells in the cell layers surrounding the vasculature and not from differentiated cells that are forced to reenter the cell cycle. This competence, however, can be manipulated. For example, leaf explants form callus on CIM only at the wound site, likely dependent on jasmonic acid action [34,56,79]. However, submergence stress releases ethylene that promotes auxin signaling, extending the callus formation response to the whole explant [80]. The ectopic expression of the auxin-induced *WOX11* gene also resulted in the extension of the responsive zone of leaf explants [76], supporting the view that auxin signaling is central in establishing competence. Furthermore, the competent cells might differ in various plant species. As described above, in Arabidopsis, certain vascular parenchyma and procambium cells in leaves, as well as the xylem-pole pericycle cells in roots, have the capability to regenerate callus [78]. In rice, however, bundle sheath cells and some immature vascular cells in leaves, as well as the phloem-pole pericycle cells in roots, have this capability [78]. Furthermore, in rice, unlike in Arabidopsis, only the OsIAA11 (the homolog of AtIAA14)-related pathway (lateral root development) but not the alternative WOX11-mediated pathway (AR development) operates during callus initiation from the primary root [67].

The other interesting finding of studying CIM-formed callus was that it is not a mass of “undifferentiated” cells but resembles an ill-organized root meristem even if it initiates from above-ground plant organs. In this respect, it is different from the callus that is formed in response to wounding without exogenous hormone application [23,41].

### 2.3. Hormone Gradients in Regeneration and Callus Initiation

Plants, as sessile organisms, face a considerable risk of getting injured and even losing whole organs or parts of them. Therefore, they have evolved molecular pathways to govern swift wound healing, tissue and organ regeneration. Plant morphogenesis, including organogenesis, strongly relies on established auxin/cytokinin hormonal gradients and interactions that need to be reestablished if disrupted by wounding in order to allow proper tissue/organ regeneration at the right location [81,82,83]. Lost single cells can be replaced by the cell-death-induced division of nearby cells, the daughters of which differentiate according to their position determined by hormone gradients as well as short-ranged cell-to-cell signaling [7,84,85]. Tissue replenishment, although triggered by short-ranged wound signals, is controlled by long-ranged hormonal gradients. In agreement, removing the root tip evokes an auxin synthesis/signaling response, while that of the shoot tip evokes a cytokinin-dependent one, allowing the regeneration of the lost meristematic tissues primarily controlled by auxin in the root and cytokinin in the shoot, respectively [7,40]. Furthermore, wounding at the hypocotyl base suppresses the cytokinin-related signaling to allow auxin-regulated processes to take place, which is in accord with the regeneration of AR meristems replacing the lost root system instead of cytokinin-mediated regeneration of lost shoot tissues [86]. Small incisions or grafts, although they disturb the hormone gradients, do not block the hormone transport completely, which ensures the controlled regeneration/reunion of tissues, including the re-establishment of the vascular connection [1,30].

Irrespective of the subsequent organ/callus regeneration pathways, wounding seems to trigger the expression of the genes in the *ERF115*/*109*-*PAT*-*WIND1* pathway both in the root and the shoot, likely via jasmonic acid signaling [1,34,35,36,37,40,43]. However, this wounding-related pathway is in a feed-forward loop with auxin signaling in the case of root regeneration [37] and cytokinin signaling in the case of shoot regeneration [43]. There are, however, situations when the normal hormone gradients cannot be directly or at all re-established. In such cases, forming a callus tissue is the first step or even the final solution for wound healing. Although wound-induced organ regeneration or callus formation can share the same initial steps, in the absence of proper hormone gradients, callus cells cannot follow a spatially regulated tissue differentiation pathway and, after stopping division, differentiate as parenchymatic cells, closing the wound. Application of exogenous auxin and cytokinin in CIM also disturbs the endogenous hormone gradients. The auxin dominance in CIM evokes the root formation response irrespective of the organ or explant [66]. This response might start as either the LR or the AR induction pathway in Arabidopsis [67]. However, due to the continuous presence of exogenous hormones, proper endogenous gradients cannot be established in the forming primordium. This results in overproliferation followed by disorganization of the tissues, although they still express proper root meristem markers, at least in a transient manner [66]. Callus cells can continuously divide and be long maintained in the presence of the proper auxin/cytokinin ratios. Although, due to intensive research, we know a lot about how callus development starts in response to wounding and/or plant hormones, our knowledge about the molecular nature of established callus cultures is scarce. Especially as these cells are generally considered “dedifferentiated,” with no specific gene expression pattern except the expression of genes required for cell division.

## 3. Molecular Signature of Callus Tissues

### 3.1. The Induction Phase

**Figure 1:**
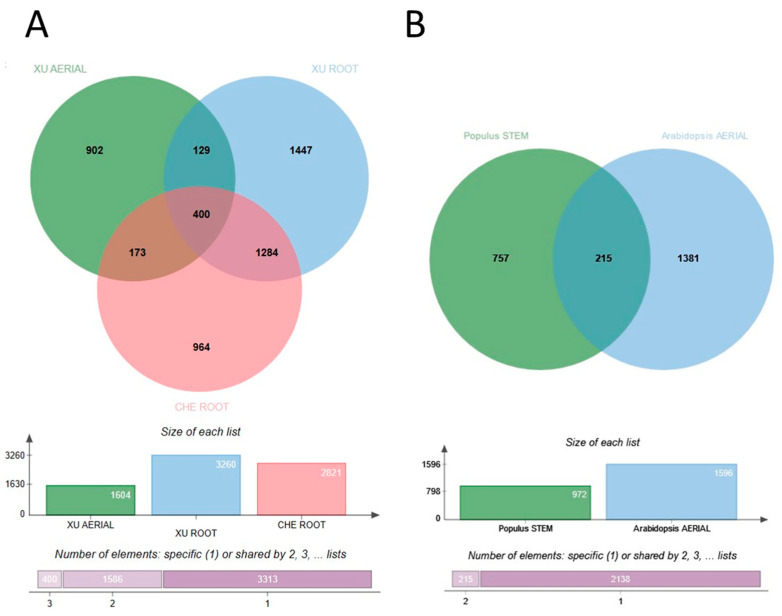
(**A**) Comparison of upregulated gene expression in Arabidopsis aerial and root explants that were placed onto CIM for four days [87,88]. (**B**) Comparison of upregulated gene expression in Arabidopsis aerial [88] and Populus stem [89] explants that were placed onto CIM for four and three days, respectively. Upregulated genes were defined in reference to gene expression in the corresponding untreated explant in each case using appropriate statistics described in the cited references. The diagrams were prepared by jvenn [90].

Wounding and exogenous hormones (i.e., CIM) evoke molecular responses that lead to the development of seemingly identical callus tissues. However, as highlighted above, the steps of the signaling pathways leading to callus formation exhibit considerable differences depending on the organ, the inducer, and the plant species. The same holds true for the molecular landscapes of the formed initial callus tissues [21]. While the CIM-induced callus exhibits a gene expression pattern resembling that of the root meristem [66], it is not the case if the callus forms in response to wounding [23,41]. In agreement, regulated genes in 35S:WIND1 seedlings exhibit limited overlap with that of an auxin(2,4-dichlorophenoxyacetic acid; 2,4-D)-induced callus [41]. Even in the case of the same callus induction method (CIM), the upregulated genes significantly differ depending on the initial explant and even from experiment to experiment (Figure 1A). Che [87] and Xu [88], with their co-workers, analyzed the gene expression of root and root or aerial tissue explants, respectively, placed onto CIM for up to four days. Although they experienced, in all cases, thousands of genes differentially expressed in the calli in comparison to the initial explants, there were only 400 common upregulated genes in the datasets (Figure 1A). As shown in Table 1, this gene set is primarily enriched in cell cycle and auxin response-related genes, but ribosome biogenesis and root system development genes are also overrepresented (for details, see Supplementary Materials File S1).

A comparison of Populus stem internode explants incubated for three days on CIM [89] to Arabidopsis aerial tissues placed onto CIM for four days [88] revealed only 215 common induced genes (Figure 1B; Supplementary Materials File S2). Among these, 108 were also differentially expressed in Arabidopsis root explants cultured on CIM for four days (Supplementary Materials File S2). Interestingly, this limited common gene set is still significantly enriched in genes implicated in ribosome biogenesis. This list, however, also includes genes coding for the AUXIN RESPONSE FACTOR 5 or MONOPTEROS (ARF5/MP) TF, the IAA13, 19, and 30 auxin-related transcriptional regulators, the GH3.1, WES1, and DWARF IN LIGHT 1 (DFL1) auxin conjugases, the auxin-induced organ patterning factors PLETHORA 3 (also named as AINTEGUMENTA-LIKE 6 or AIL6), LATERAL ROOT PRIMORDIUM1 (LRP1), TORNADO 2 (TRN2), and WRKY DNA-binding protein 23 (WRKY23). This pattern highlights extensive auxin-related protein synthesis activity likely related to an early phase of cellular reorganization. Cell cycle-related genes are missing from the common gene set due to the one-day-earlier time point (3rd day) in the case of Populus than in Arabidopsis (4th day). In the case of Arabidopsis explants, cell cycle genes were only detected as differentially expressed genes (DEGs) at 4 days but not at 2 days after CIM incubation of explants [88].

All these experiments were focused on the initial events of callus induction by CIM and, not surprisingly, showed the upregulation of auxin-related molecular pathways, which likely differentially affected downstream genes depending on the type and condition of the explant. This obviously resulted in highly divergent gene expression patterns. From these studies, there is no clue to answer the question of whether established callus tissues formed from different explants in response to various inducers share a common gene expression pattern that can define them as callus tissue.

### 3.2. Young Calli

**Figure 2:**
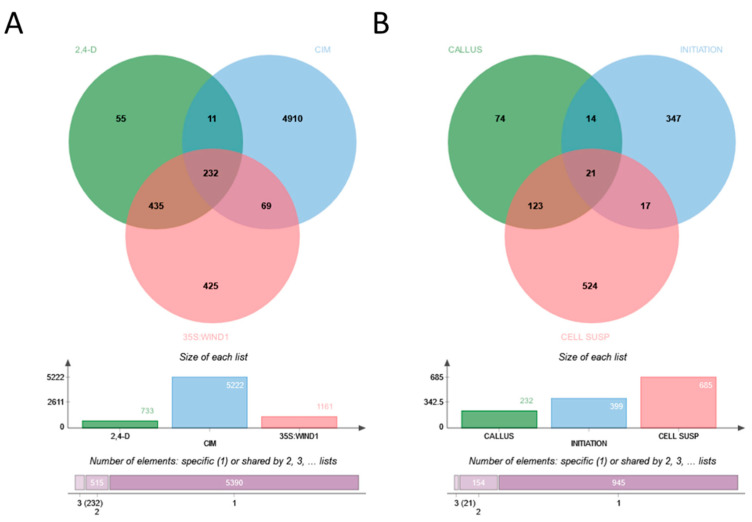
(**A**) Overlap among upregulated genes in two-week-old hormone- and wound(35S:WIND1)-induced calli, respectively. CIM–DEGs at least 2-fold upregulated in leaf-derived calli induced by CIM [93]; 2,4-D–DEGs at least 2-fold upregulated in calli developed on 2,4-D-induced seedlings [41]; 35S:WIND1–DEGs at least 5-fold upregulated in calli that autonomously developed on 35S:WIND1 transgenic seedlings [41]. (**B**) Overlap among upregulated genes in two-week-old callus tissues (CALLUS; Supplementary Materials File S3), in 4-day-old CIM-induced explants (INITIATION; Supplementary Materials File S1), and a long-term cell suspension culture (CELL SUSP; [41]). Upregulated genes were defined in reference to gene expression in the corresponding untreated explant/seedling in each case using appropriate statistics described in the cited references. The diagrams were prepared by jvenn [90].

An initial attempt to compare gene expression in cultured cells to that of seedlings revealed downregulation rather than induction of gene expression in agreement with losing differentiated cell functions during in vitro culture [92]. Nevertheless, this approach led to the first recognition of the WIND1 TF as a “dedifferentiation” factor [92]. A more detailed follow-up analysis focusing on the role of WIND1 confirmed this early finding but provided a more detailed view of differential gene expression in callus tissues [41]. In this study, DEGs in wound-(35S:WIND1) and auxin-(2,4-D) induced two-week-old calli were identified in reference to gene expression in uninduced seedlings [41]. In this way, several hundred genes could be determined, the upregulation of which in callus cells was independent of the way of induction. Among these genes, 21 codes for AP2/ERF domain TFs including the regeneration-related WIND1, ESR1, Rap2.6L, ERF114, PLT3, 5 and 7. Lee and their colleagues reported differential gene expression in two-week-old CIM-induced calli developing on leaf explants [93]. The overlap among the DEGs in these calli (5708 genes) and the strong callus upregulated gene list of Iwase and their colleagues (658 genes; upregulated both in 35S:WIND1- and 2,4-D-induced calli) is rather limited, highlighting only 232 genes (Figure 2A; Supplementary Materials File S3).

Interestingly, this common list is enriched in genes expressed in response to stress, especially hypoxia (Table 2). This may reflect the stressful in vitro conditions and the oxygen-limited environment inside the growing calli. This is supported by the fact that among the hormone-responsive DEGs, those regulated by abscisic acid and/or jasmonic acid are more prevalent in this list than those controlled by auxin (Supplementary Materials File S3). Nevertheless, the auxin-responsive *ARF5/MP* was present in all the above datasets, such as the regeneration-related ones, *Rap2.6L*, *ERF114*, *PLT3*, and *PLT5*, while *WIND1* was not among the DEGs in the leaf callus study. Of note is the high number of WRKY and NAC TF genes also differentially expressed in all the above-investigated calli. Interestingly, if we compare the 400 DEGs obtained four days after 2,4-D-induction (at the time of first cell divisions) with the 232 DEGs of young calli (two weeks after induction), there is an overlap of only 35 genes (Supplementary Materials File S3), indicating a continuing reorganization of the gene expression pattern during the first weeks of callus establishment.

To confirm the association of the expression of these genes with early callus development, I used the dataset of Xu et al. [94], who measured gene expression during the first 30 days of Arabidopsis protoplast culture. The protoplasts were cultured in liquid media, and the protoplast induction medium (PIM; with 2,4-D and thidiazuron as hormones) was changed to the callus induction medium (CIM1; thidiazuron only) at 11 days. Division of the protoplast-derived cells started at 4 days, and microcalli were present from 11 days onward.

**Figure 3:**
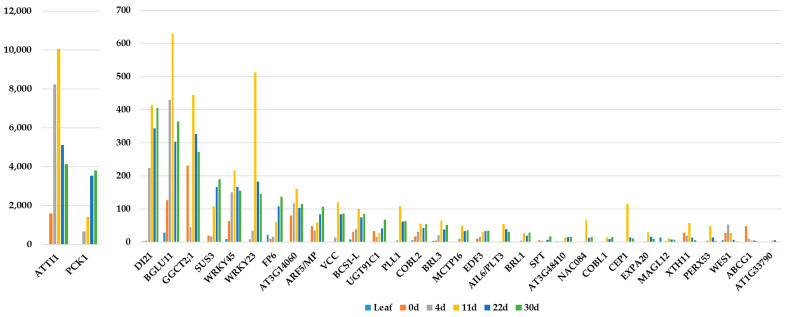
Expression of 33 callus-related genes during the development of protoplast-derived microcalli. The values are the average reads per kilobase of transcript per million mapped reads (RPKM) and were extracted from the data published in [94]. For the full name/description of the genes, consult Supplementary Materials File S3.

In Figure 3, the expression of 33 out of the 35 genes is shown. Two genes, AT1G62763 and AT5G26665, although they were represented on the Arabidopsis microarrays, are now not among the genes of the TAIR10 genome assembly of Arabidopsis (https://www.arabidopsis.org/, accessed on 27 July 2023). Most of the 33 genes exhibited increasing expression in the protoplast-derived microcalli, with a peak at 11 days, at the time when PIM was changed to CIM1. Despite a drop in the gene expression level afterward, the transcript levels were still higher in the growing calli than in the leaves or the freshly isolated protoplasts for the majority of genes.

### 3.3. Established Callus Cultures

**Figure 4:**
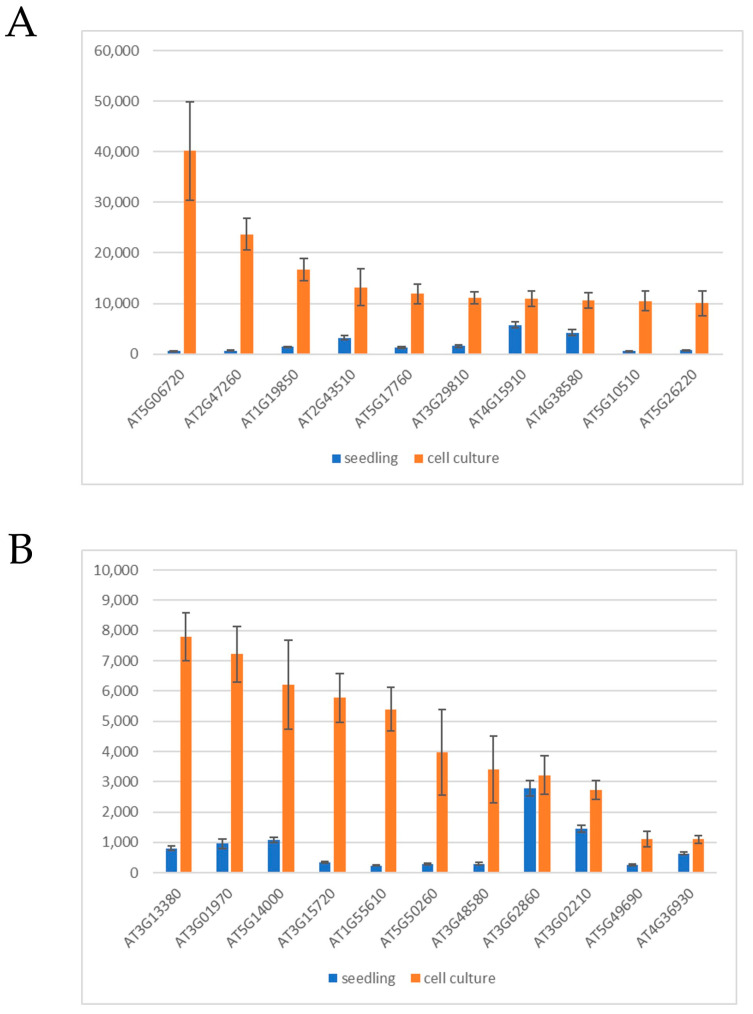
(**A**,**B**) Average signal intensity differences with standard errors from ten-ten microarray hybridization experiments (AT_AFFY_ATH1-6) investigating gene expression in seedlings and cell cultures, respectively. Only wild-type untreated samples were considered with all repetitions (altogether, 23 data for both types). Values (averages as well as SE) were generated by the Genvestigator tool [95]. The genes were divided into two graphs according to signal strength. All genes were classified by Genevestigator as having high or moderate gene expression in cell cultures. The description of source experiments and detailed data with confidence intervals are supplied as Supplementary Materials File S5.

The previously mentioned gene expression investigations were carried out with “young” (two weeks old) calli developing on explant surfaces. Calli, however, can be maintained as cell suspension cultures for a long time in the presence of auxin and cytokinin. It is, therefore, a valid question how the initial gene expression pattern of callus cells is preserved during long-term culture. Iwase and colleagues [41] compared the DEGs in 35S:WIND1 and 2,4-D-induced calli to those of the T87 cell suspension culture (all DEGs were defined in comparison to seedlings). Out of the 232 upregulated DEGs of 35S:WIND1 and 2,4-D-induced calli, 144 were also upregulated (at least two-fold) in the T87 cell culture (Figure 2B; Supplementary Materials File S4). This gene set is enriched in stress-related genes, especially those responding to hypoxia, as well as in hormone-responsive ones (Table 3). The gene list includes the genes of some of those factors that were implicated in the control of regeneration processes, including the ARF5/MP, PLT3/AIL6, RAP2.6L, and the WRKY23 TFs. Among the upregulated DEGs of callus induction, in young calli and in established cell cultures, there are 21 that are common (Figure 2B, Table 4). To confirm the preferential expression of these genes in cultured cells, not to rely only on the single experiment of [41], two gene expression datasets of untreated, wild-type seedlings and cell cultures, respectively, were assembled from ten-ten microarray experiments (AT_AFFY_ATH1-6) randomly selected from those stored in the Genvestigator database [95]. As shown in Figure 4, except for one (AT3G62860), all 21 genes exhibited higher absolute expression levels in the cell cultures than in seedlings, although at different expression strengths and ratios.

## 4. What Can Callus-Expressed Genes Teach Us?

Although stress-related genes are prevalent in all callus-related gene expression datasets, and one would expect cell division-related genes to be upregulated in all callus tissues that have dividing cells, neither strictly stress- nor cell division-associated genes are present in the common gene set of callus cells (Table 4). As the callus grows, its outer and inner cell files face different metabolic and hormonal conditions and can exhibit differences in gene expression, including the expression of a variety of stress-related genes identified in all callus transcriptomes. However, the expression of these genes is highly variable in time as well as from condition to condition, and most likely, this is the reason that they are not strictly related to the callus cell fate. Cell cycle genes are among the genes that are upregulated in callus cells only at the initiation phase, at the time of their first division but are not among the upregulated genes in larger growing cell colonies. The reason can be that while the first cell divisions are still more or less synchronous, at later stages, cell division is restricted to the edge of the callus tissue and proceeds in an asynchronous way limiting the detection of cell cycle-related gene activity.

Importantly, however, the list of 21 genes contains six TF genes, one of which is the auxin response factor *ARF5/MP* gene, a key regulator of cell specification during both embryogenic and post-embryogenic development. *ARF5/MP* is expressed in the lower tier of the globular embryo, and at the heart stage, its promoter is active in the provascular tissue [96]. It was shown that, during Arabidopsis embryogenesis, ARF5/MP cell-autonomously controls vascular and ground tissue initiation, while root meristem specification depends on non-cell-autonomous *ARF5/MP* action [97]. In agreement, the *mp* mutation corrupts the formation of the body axis as well as the vascular strands during embryogenesis, and the *mp* seedlings are rootless [98]. In the primary root, *ARF5/MP* expression is confined to the QC and the fast-dividing cells around it [96]. *ARF5/MP* is also expressed during LR initiation and meristem formation [99]. *ARF5/MP* expression is already detected in the daughter cells of the LR founder cells originating in the pericycle [100]. In the emerging lateral root primordia (LRPs), *ARF5/MP* expression was prominent in the central cell files and excluded from the flanks [100,101]. LR initiation is controlled by two auxin response modules, the SLR/IAA12-ARF7 and ARF19, and the BDL/IAA12-ARF5/MP modules, which act in sequential order [102]. At a later stage, the two modules mutually inhibit each other, confining *ARF5/MP* (as well as *ARF6* and *8*) expression to the central meristematic domain and *ARF7* expression to the flanks where cell division stops [101]. In the developed LR meristem, *ARF5/MP* expression is restricted to the columella and the central cylinder, as in the primary root [96]. ARF5/MP is also present in the shoot apical meristem (SAM), not only in the dividing cells of the meristem but also in the differentiating cells of the lateral organ primordia [99,103,104]. In the center of the SAM, ARF5/MP contributes to the stability of the stem cell pool, promoting *WUS* expression via the repression of *DORNROSCHEN/ENHANCER OF SHOOT REGENERATION 1* (*DRN/ESR1*) and *ARR7/ARR15* genes and thus negatively affecting *CLAVATA3*(*CLV3*) [103,104]. Interestingly, in the embryo, ARF5/MP has an opposite (activating) role in regulating the same genes [103,104,105]. At the periphery of the SAM, ARF5/MP non-cell autonomously controls PIN1 polarity, promoting the self-regulated establishment of local auxin maxima, further strengthening *ARF5/MP* expression that subsequently triggers primordium development [106]. Thus ARF5/MP contributes to the stabilization of cell fates in distinct zones of the SAM [107]. The dominant allele of *ARF5/MP* causes severe vascular disorganization in lateral organs and disrupts the adaxial-abaxial polarity [108]. Altogether, ARF5/MP is an important regulator of ground, vascular, and stem cell identities, meristem maintenance and function, and the initiation of lateral organ primordia.

Considering the similarity of LRP initiation and callus formation [66], it is not surprising that *ARF5/MP* expression was also studied during the latter process [109]. The *ARF5*/*MP* gene was activated in the pericycle cells all over the explant already at 12 h after placing root explants on CIM. ARF5/MP remained abundant in the dividing and then rapidly proliferating pericycle-derived cells forming the callus. In the developing callus, it was first limited to the growing front, and then its level gradually diminished, unlike in emerging LRPs, where it was continuously confined to the central meristematic region. Placing the calli on a shoot-inducing medium (SIM), ARF5/MP is detected at the periphery of forming shoot primordia [109]. A constitutively active variant of ARF5/MP promoted shoot organogenesis [109], while its inducible expression was sufficient to revive auxin-mediated patterning processes in completely disorganized shoot as well as root tissues [110]. ARF5/MP exerts its tissue patterning effect by controlling, among others, the genes of the auxin efflux carrier PIN proteins [110]. In summary, ARF5/MP is a good candidate to maintain the auxin-dependent cell division activity as well as the organogenic potential of calli.

The TF PLETHORA3 (PLT3) can have similar importance and role in the callus cell fate. PLT3 belongs to the AINTEGUMENTA-LIKE (AIL) TF family and is also designated as AIL6. Members of the AIL family are known to control embryogenesis, meristem development, and lateral organ initiation in concert with auxin regulation [111]. *AIL6/PLT3* expression is undetectable during early embryogenesis, but at later stages, it is confined to the QC and the surrounding cells, including the ground tissue and provascular cells [112,113]. In agreement, the *plt1;plt2;ail6/plt3* triple mutant embryos have an aberrant embryonic root pole, and their seedlings are rootless [112,113]. In the primary root, *AIL6/PLT3* expression is highest in the columella stem cells, while that of *PLT1*, *PLT2*, and *PLT4/BABY BOOM*(*BBM*) the key regulators of post-embryogenic root development is characteristic for the QC [112,113]. During LR formation, PLT3, PLT5, and PLT7 redundantly control the first events, including the first formative cell divisions and the establishment of growth polarity. Their action is a prerequisite for the subsequent specification of the LR meristem, controlled by PLT1, PLT2, and PLT4 [114]. Consequently, LRP development is seriously impaired in the *ail6/plt3 plt7* double mutant [115].

AIL6/PLT3, together with AINTEGUMENTA (AINT) and PLT7, play a significant role in the maintenance and functioning of the shoot apical meristem as well [116]. *AIL6/PLT3* is expressed in the center as well as in the periphery of the SAM, indicating its role both in meristem maintenance and lateral organ formation in the shoot, such as in the root [116,117]. Indeed, AIL6/PLT3 controls the site of lateral organ initiation (phyllotaxis) in the SAM [115,116,117,118]. The auxin-dependent initiation of flower primordia is also dependent on the redundant action of AIL6/PLT3 and ANT [119,120,121]. AIL6/PLT3 was shown to exert its action during meristem maintenance and organ initiation by controlling auxin synthesis and response, cell division, and cell wall organization [101,118,121,122].

It is obvious that ARF5/MP and AIL6/PLT3 control very similar processes during plant development. Nevertheless, there is only limited data available about their interlinked action. During LRP initiation, *AIL6/PLT3* expression in the pericycle and pericycle-derived cells is indirectly controlled by the early-acting SLR/IAA12-ARF7 and ARF19 auxin response module [115]. However, at later stages, *AIL6*/*PLT3* expression is confined to the central region of dividing cells, where ARF5/MP is active and excluded from the flanks controlled by ARF7 [101]. During flower primordium initiation, ARF5/MP directly controls *AIL6/PLT3* (and *ANT*) expression [123], and they parallel activate the gene coding for LEAFY (LFY), one of the flower meristem identity factors [124].

Considering the overlapping key roles of ARF5/MP and AIL6/PLT3 in plant growth, organ formation, and tissue patterning, it was obvious to ask the question of whether the other nineteen callus genes also have related functions, despite their seemingly non-related functional annotations.

The SPATULA (SPT) bHLH family TF (AT4G36930) was originally annotated to regulate pistil development and seed dormancy [125,126]. However, it is now clear that it has a much wider impact on plant development. Among other things, it was shown that the loss of SPT function results in an increased QC size in both embryonic and post-embryonic roots and leads to the development of longer primary roots [127]. SPT might affect QC size depending on auxin transport and accumulation [127]. In agreement with its role in controlling QC size, *SPT* expression was used as an embryogenic root stem cell marker [128]. Interestingly, local inhibition of ARF5/MP activity in the first embryonic vascular and ground tissue cells resulted in the downregulation of the *SPT* gene indicating that its expression in these cells is ARF5/MP dependent [97]. However, the *SPT* gene is not directly regulated by auxin [129]. Transcriptional interaction between SPT and the PLT4/BBM factor on root growth was indicated by the phenotype of the double mutant [130]. Other PLT TFs, including AIL6/PLT3, were capable of upregulating the *SPT* gene in the growing root [131]. SPT also controls leaf size [132] and is expressed at the proliferative front of leaf primordia [129,132]. The meristematic region of leaf primordia is increased in the *spt* mutant, indicating that it negatively controls cell divisions [132]. SPT also inhibits cotyledon expansion under the control of DELLA transcriptional repressors [133]. Altogether, it seems that SPT might have a general role in limiting meristem and primordium size during plant development [134].

Members of the WRKY TF family are considered to be key regulators of plant defense and adaptation, although accumulating evidence highlights their importance in plant developmental processes [135]. WRKY23 (AT2G47260) was first described as a TF involved in the plant defense against nematodes [136], but later, somewhat unexpectedly, it turned out to play a significant role in auxin transport. It was found that the *WRKY23* gene is auxin-regulated [137,138,139] and its loss- or gain-of-function mutations interfered with the transcriptional feedback control of auxin’s own transport [139]. WRKY23 controls PIN protein localization and, thus, auxin transport polarity [137,139]. In agreement, WRKY23 was shown to be involved in a variety of developmental processes dependent on proper auxin transport. During embryogenesis, *WRKY23* expression is confined to the shoot and root stem cell niches, and the loss of its function prevented root pole specification and meristem establishment [138]. In the embryo, *WRKY23* is under the control of the IAA12/BDL-ARF5/MP auxin response module [138]. In the post-embryonic root, WRKY23 contributes to the maintenance of the root stem cell niche in a dosage-dependent manner under the control of the *SLR*/*IAA12*-*ARF7* and *ARF19* auxin signaling pathway [137]. *WRKY23* is also expressed in the SAM and the prevascular and vascular cells of flowers, cotyledons, and leaves [139]. In these cells, WRKY23 exerts its effect on PIN-dependent auxin canalization, promoting the expression of genes coding for the CANALIZATION-RELATED AUXIN-REGULATED MALECTIN-TYPE RECEPTOR-LIKE KINASE (CAMEL) together with CANALIZATION-RELATED RECEPTOR-LIKE KINASE (CANAR), which phosphorylate the PIN auxin transporters [140]. WRKY23 was also hypothesized to control the auxin transport indirectly by augmenting flavonol biosynthesis [137,139].

The other callus-expressed WRKY TF, WRKY45, was mainly studied with respect to its role in leaf senescence and defense responses [141,142,143,144]. However, it was also found to be part of the transcriptional network controlling vascular development in Arabidopsis [145]. A yeast one-hybrid assay revealed that WRKY45 binds the promoter of *IAA20/BDL*, the repressor of ARF5/MP, as well as the promoters of the genes of the HD-ZIP III TFs, PHABULOSA (PHB) and REVOLUTA (REV) [146,147]. PHABULOSA was shown to directly bind the promoters of both *ARF5/MP* and *IAA20/BDL* and modulate the auxin response during vascular patterning [146].

Only limited information is available about the NO APICAL MERISTEM (NAM) family protein NAC DOMAIN CONTAINING PROTEIN 84 (ANAC084; AT5G14000) of Arabidopsis. Some members of the NAM family, as the name of the family indicates, have been implicated in meristem formation, but their functions are rather diverse in agreement with the large size of this plant-specific TF family [148]. The *ANAC084* gene is regulated in both the *AtMYB59* mutant and overexpressing lines [149]. *AtMYB59* is strongly expressed in the root and controls cell division in the root meristem [149]. *NAC084* is upregulated in the *hub1-1* mutant; the HISTONE MONOUBIQUITINATION1 (HUB1) RING E3 ligase protein also has a role in cell cycle regulation and organ growth [150]. Furthermore, A*NAC084* expression in the SAM is controlled by PLT3 [121,131]. Altogether, this limited information suggests that the function of ANAC084 might be associated with cell cycle control in meristematic tissues.

Besides the above TFs, the expression of two receptor-like kinase (RLK) genes, those of *BRI1-LIKE1* (*BRL1*; AT1G55610) and *BRL3* (AT3G13380), also characterizes the callus cells. These leucine-rich repeat RLKs are close homologs of the BRASSINOSTEROID INSENSITIVE1 (BRI1) RLK and are preferentially expressed in vascular tissues all over the plant, but *BRL1* is rather expressed in more mature cells than *BRL3* [151]. In agreement, the *bri1 brl1 brl3* triple mutation exacerbated the defects in the vascular phenotypes of the *bri1* mutant [151]. At the root apex, BRL1 and BRL3 were both observed in the QC, columella stem cells, and a cluster of provascular cells, including vascular initial cells situated directly above the QC [152]. While BRI1 levels are low in the QC and the provascular cells and high in the outer tissue layers of the primary root, the distribution of BRL1 and BRL3 is the opposite [152]. It seems that BRL3 is excluded from the outer root cell layers due to post-transcriptional modifications. Based on these observations, it is supposed that while BRI1 signals from the epidermis control root meristem size [153], the BRLs have the role of perceiving and mediating brassinosteroid signals in the innermost root tissues [152]. In agreement, besides BRI1, the BRL1/BRL3 receptor complex also contributes to QC organization and root growth [152]. It is of note that *BRL3* is among the genes downregulated by the *ant ail6/plt3* mutation in the SAM [124].

**Figure 5:**
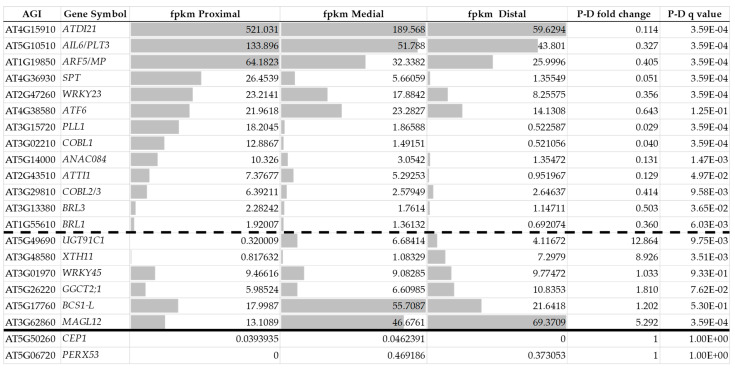
Expression of the 21 callus-related genes in the proximal, medial, and distal regions of the Arabidopsis root meristem, respectively. The dashed line separates the genes more strongly expressed in the proximal than in the distal domain from those showing the opposite pattern. The solid line separates the two genes with very low transcript levels in all regions. The data were extracted from [191]. “fpkm”: fragments per kilobase of transcript per million mapped reads. P-D: the difference between proximal and distal values.

**Figure 6:**
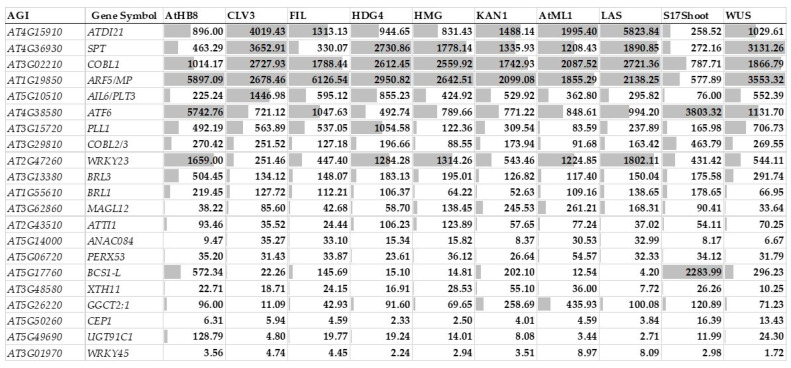
Expression of the 21 callus-related genes in the shoot apical meristem at different developmental domains indicated by marker genes. The data were extracted from [192]. AtHB8: Shoot xylem/vasculature cell type; CLV3: Central zone/stem cells; FIL: Organ primordia/peripheral zone cell type; HDG4: L2 layer/subepidermal cell type; HMG: Meristematic L1 layer/epidermal cell type; KAN1: Abaxial organ boundaries/peripheral zone cell type; AtML1: Ubiquitous L1 layer/epidermal cell type; LAS: Adaxial organ boundaries/peripheral zone cell type; S17shoot: Shoot phloem/vasculature cell type; WUS: L3 layer/rib zone cell type.

The AT4G15910 gene product was annotated as DROUGHT-INDUCED 21 (ATDI21, DI21). DI21 is a member of the LATE EMBRYOGENESIS ABUNDANT PROTEIN (LEA) family (and is also designated as LEA41). These small proteins are primarily implicated in cellular dehydration tolerance during development as well as in response to stress [154]. However, their function is likely much wider than that. The high-level expression of the *DI21* gene, for example, strongly correlates with the expression of the meristem cell fate markers *PLT1* and *PLT4* in the stem cell cluster of the Arabidopsis root [155]. The Arabidopsis RNA-BINDING PROTEIN1 (AtRBP1) that is produced in actively proliferating cells of Arabidopsis [156] was shown to bind the mRNA of *DI21 in planta* [157]. *DI12* is strongly expressed in the root and shoot meristem (Figure 5 and Figure 6). Furthermore, the gene is expressed in the provascular and procambial cells of the Arabidopsis leaf [158]. It was found to be upregulated by ectopic *CLE41* expression [145]. CLE41 is the precursor of the peptide ligand for the PHLOEM INTERCALATED WITH XYLEM/TDIF RECEPTOR (PXY/TDR) controlling the vascular cambium. Therefore, *DI21* expression seems to be strongly associated with meristematic cell fate.

Members of the pectin lyase-like (PLL) superfamily have various functions associated with pectin degradation and cell separation [159]. The PLL1 protein coded by the AT3G15720 gene has not been characterized in detail, but its name has appeared in many transcript analysis studies implicating its wide role in developmental processes. Among others, it was in the gene set exhibiting expression in provascular and procambial but not in mesophyll or guard cells of the Arabidopsis leaf [158]. In agreement, the gene was upregulated by *CLE41* expression, which controls the cambial cell fate [145]. Its expression in vascular initials was confirmed by in situ hybridization in Arabidopsis embryos as well [160]. Its transcripts are enriched in root QC cells [161]. The *PLL1* gene is regulated by the SHOOT MERISTEMLESS (STM) TF [162], the ANT and AIL6/PLT3 TFs [121] in the SAM, and also by the RAPL2.6L TF, which is implicated in shoot regeneration [87].

The XYLOGLUCAN ENDOTRANSGLUCOSYLASE/HYDROLASE11 (XTH11; AT3G48580) protein also belongs to a large enzyme family controlling cell wall structure [163]. Specific functions of XTH11 are unknown. Its expression was found to be five-fold upregulated in the Arabidopsis *scz* mutant [164]. The SCHIZORIZA (SCZ) TF was implicated in stem cell formation and cell fate separation [165]. Moreover, the expression of the *XTH11* gene could be detected in isolated provascular–procambial cells of the leaf [158].

The function of the COBRA-LIKE1 (COBL1) and COBL2 proteins is also associated with cell wall structure since COBRA (COB) and its family members are implicated in the deposition of crystalline cellulose into the cell wall during cell expansion and differentiation [166,167]. Considering their tissue-specific expression, *COBL1* was shown to be expressed in the root columella and stele, in developing LRPs, and in the vascular tissue of the leaf, while *COBL2* was demonstrated to be active in the root columella, in the root cells through which the LRP breaks out, and in the vasculature of sepals, at the surface of the stigma, and in the ovules, and also at the tip of the embryonic root [168]. In the *ant ail6/plt3* mutant SAM, *COBL2* expression is 1.5-fold upregulated [121].

The AT5G06720 gene codes for the class III peroxidase AtPRX53 (also annotated as ATPA2), and it has been reported to be involved in plant-nematode interaction and cell elongation as well as cell wall lignification [169,170]. The gene is regulated by brassinolide but not by auxin [171] and is expressed in the middle sections of primary stems [172]. Promoter:GUS fusion experiments highlighted its strong expression in the root vascular tissue as well as at the sites of LR emergence [169,170]. The *AtPRX53* gene is among the genes regulated by the LBD11 TF [173]. LBD11 controls vascular cambium activity through the balanced generation of reactive oxygen species [173]. Strengthening its role in vascular development, the *AtPRX53* gene is also upregulated by the VASCULAR-RELATED DOF1 (VDOF1) and VDOF2 TFs, which are known to control lignin biosynthesis and vascular cell differentiation in Arabidopsis [174]. Furthermore, its expression was detected in isolated provascular–procambial cells [158] but was found to be downregulated by ectopically expressed *CLE41* [145].

The gene AT5G26220 coding for the ChaC-like family protein GAMMA-GLUTAMYL CYCLOTRANSFERASE 2;1 (GGCT2;1) has a role in glutathione (GSH) degradation, and its expression is induced by various stress treatments and especially by sulfur starvation [175]. *GGCT2;1* expression is strongly induced by S-starvation in the primary root tip. The enzyme activates the γ-glutamyl cycle that contributes to changes in the root system architecture, promoting root growth but suppressing LR formation [175]. AIL6/PLT3 can upregulate *GGCT2;1* expression in the root [131].

The defensin-like protein coded by AT2G43510 is annotated as TRYPSIN INHIBITOR PROTEIN 1 (ATTI1, TI1). The gene can serve as an oxidative stress marker, as it is quickly and strongly activated in response to various stress treatments [176]. That the gene might have developmental roles as well is indicated by its activation during leaf senescence [177], in response to gravity stimulation [178], in the S-phase of the cell cycle [179], and in response to the ectopic expression of *CLE41* [145]. In agreement, it has been found to be expressed in isolated provascular–procambial cells [158].

The stress-induced AT4G38580 gene codes for the FARNESYLATED PROTEIN 6 (ATFP6), which has a heavy metal-associated domain and is also annotated as HEAVY METAL-ASSOCIATED ISOPRENYLATED PLANT PROTEIN 26 (HIPP26) [180]. The gene is strongly expressed in the vascular tissue [180] and provascular–procambial cells [158].

Our knowledge about the gene AT3G62860, which codes for the MONOACYLGLYCEROL LIPASE 12 (MAGL12) protein, is scarce. It has been shown to be ubiquitously expressed in Arabidopsis organs [181], including procambial–provascular cells [158].

The UDP-GLYCOSYLTRANSFERASE 91C1 (UGT91C1) coded by AT5G49690 was found to confer triketone herbicide resistance [182] and was shown to be strongly induced by berberine, which causes leaf polarity defects [183]. Its expression is under the control of the VASCULAR-RELATED NAC-DOMAIN 7, which directly regulates the expression of genes for xylem vessel formation [184], and *UGT91C1* is expressed in laser-dissected procambial–provascular cells [158].

The CYSTEINE ENDOPEPTIDASE 1 (CEP1; AT5G50260) is a papain-like cysteine protease. The Arabidopsis cysteine proteases AtCEP1, AtCEP2, and AtCEP3 have been implicated in the collapse of tissues during programmed cell death (PCD) [185,186]. In addition to their role in PCD, their functions in root development have also been reported [187]. It was found that the loss of *AtCEP2*, but not *AtCEP1*, resulted in shorter primary roots due to a decrease in cell length. *AtCEP1* and *AtCEP2* were expressed in separating root epidermal cells at the site of LR emergence, and the loss of either *AtCEP1* or *AtCEP2* caused a delay in the emergence of LR primordia. These findings suggest that these cysteine proteases are involved in developmental cell elongation and cell separation processes dependent on cell wall remodeling. In agreement, it was demonstrated that CEPs process the extensin proteins, which control cell wall structure and cell expansion [188].

AT5G17760 codes for an uncharacterized AAA+ ATPase [189], the BCS1-LIKE PROTEIN of Arabidopsis. These proteins are molecular chaperones that support protein folding and unfolding, aid the formation or disassembly of protein-protein or DNA–protein complexes, assist membrane fusion, help to unravel the DNA, and take part in protein degradation. BCS1-LIKE PROTEINs may act as floral repressors [190]. It was among the genes that were expressed in laser-dissected procambial–provascular cells [158].

Most of the above 21 genes can be somehow linked to meristem, lateral organ, and/or vascular development, respectively, although at different levels. To confirm this observation, the expression of the 21 genes was investigated in root apex and shoot meristem gene expression datasets. Wendrich et al. (2017) [191] demonstrated that development-related genes were strongly expressed in the proximal region of the meristem, and their expression gradually decreased towards the distal regions, whereas cell differentiation-related genes displayed an opposite gradient. They used the activity of the *SPT* gene as one of the markers to define the proximal, middle, and distal gene expression regions. Figure 5 shows the average absolute expression values of the 21 callus genes in these three regions. One can observe that 19 out of the 21 genes are expressed in the root tip, while the expression of two of the genes could hardly be detected in the samples. Thirteen genes exhibited strong expression in the proximal meristematic region with a gradual decrease towards the distal cell differentiation zone, whereas six of the genes exhibited the opposite pattern or had a rather uniform expression in all three zones.

**Figure 7:**
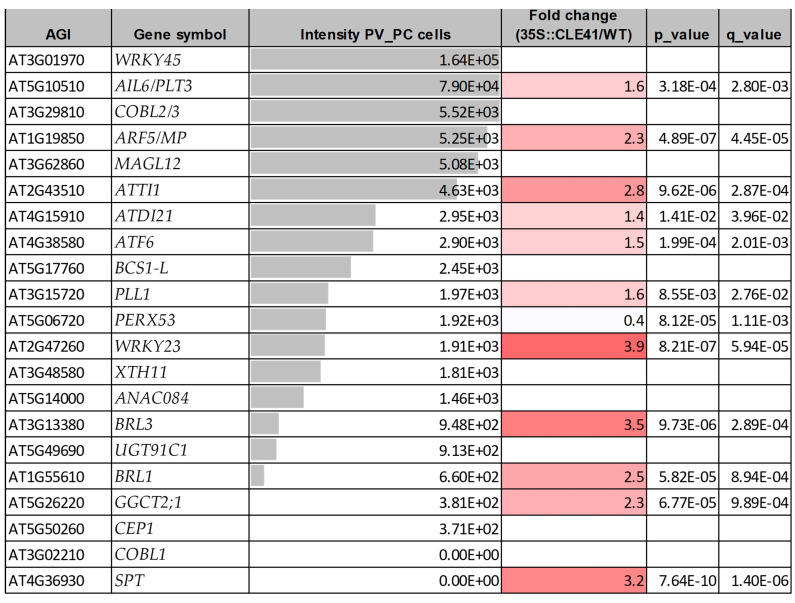
Expression of the 21 callus-related genes in provascular (PV) and procambial (PC) cells and in response to ectopic CLE41 expression. The data were extracted from [145,158], respectively. PV and PC cells were laser captured, and their transcriptome was analyzed, determining averaged signal intensities via hybridization to the Agilent 22 K Arabidopsis microarray [158]. Gene expression difference (fold change with *p* and q values) was determined among 5-week-old wild type and 35S:CLE41 transgenic Arabidopsis plants via hybridization to ATH1 Affymetrix GeneChip oligonucleotide arrays [145].

Yadav and colleagues (2014) [192] established a detailed gene expression map for the various cell types within the shoot apical meristem. In Figure 6, it can be seen that transcripts were detected in the SAM for all 21 genes, although some of them, especially the genes coding for WRKY45 and CEP1, had rather low values. Most of the genes showed expression throughout the SAM, while *BCS1-L* exhibited a phloem-specific expression. The genes strongly expressed in the root meristem region (Figure 5) were also relatively strongly expressed in the cell types of the SAM (Figure 6). These expression patterns are consistent with the data obtained during the single-cell transcriptomic analyses of the root and shoot apical meristems, respectively [193,194,195]. Furthermore, five of the genes *PLL1*, *NAC084*, *COBL1*, *GGCT2;1* and *SPT*, in addition to *AIL6/PLT3* itself, were shown to be upregulated by ectopically expressed *AIL6/PLT3* [131]. It can, therefore, be stated that the genes coding for *ATDI21*, *AIL6/PLT3*, *ARF5/MP*, *SPT*, *WRKY23*, *ATF6*, *PLL1*, *COBL1*, *ANAC084*, *ATTI1*, *COBL2/3*, *BRL3*, and *BRL1* are expressed in either the root or the shoot meristem or both. Moreover, 19 out of the 21 genes (with the exception of *COBL1* and *SPT*) were shown to be expressed in laser dissected procambial–provascular cells [158] and 11 (including *SPT*) were upregulated in 35S:CLE41 plants overexpressing the gene coding for the tracheary element differentiation inhibitory peptide CLE41 controlling the cambial cell fate [145] (Figure 7). In summary, all 21 genes are expressed in at least one of the cell types, meristematic, primordial, or provascular, with high cell division activity and developmental potency. Nevertheless, the four TFs ARF5/MP, AIL6/PLT3, WRKY23, and SPT, all having roles in spatial and temporal regulation of meristem and organ primordium initiation (including the formation of the vasculature) and maintenance, deserve special attention. The strong expression, especially of *ATDI21*, but also of *ATF6*, *COBL1*, and *PLL1* in various meristematic cell types, should prompt us to investigate their cellular functions in more detail.

## 5. What Is a Plant Callus?

The origin and nature of the plant callus tissue is controversial. It can be formed from various tissues, under various inducing conditions, and in various ways. The commonality in all pathways is that exogenous or endogenous hormones, especially auxin and cytokinin, evoke cell divisions continuing in a more-or-less unorganized way, depending on the conditions. The initial events are, however, not specific for callus formation since callus formation is a deviation from developmental organ formation or tissue regeneration pathways. The best example is callus formation from root explants. Under normal development, endogenous auxin maxima in the sensitive pericycle cells trigger LR development. Exogenous auxin can evoke the same initial response. However, under normal developmental conditions, the self-regulating auxin transport and signaling machinery spatially and temporally control organ development establishing separated developmental domains. The region where the new meristem forms is separated from the non-dividing flanks of the primordium as well as from differentiating zones of the growing organ. It was demonstrated that two antagonistic auxin response modules act during LRP organogenesis [101,102]. The SLR/IAA12-ARF7 and ARF19 and the BDL/IAA12-ARF5/MP modules mutualistically inhibit each other limiting *ARF5/MP* expression to the central pre-meristematic domain, and *ARF7* expression to the non-dividing flank. This interaction is auxin concentration-dependent: high auxin concentration leads to the degradation of BDL in the center of the primordium to allow ARF5/MP action, while at the flank, auxin concentration is low, and ARF5/MP is inactive [101]. In the case of exogenous auxin application, although the initial events are rather similar, the developmental domains cannot properly establish due to the more-or-less uniform distribution of high auxin all over the emerging primordium. In agreement, in CIM-induced roots, *ARF5/MP* expression was observed in all pericycle-derived cells that continued to divide rapidly [109]. Although the forming “callus” at the early stage expresses root meristem markers in a partly organized way resembling that of the LRP [66], in the absence of proper auxin gradients, the expression of the root meristem genes gradually decreases; a root meristem is not established. Instead, *ARF5/MP* expression is confined to the front maintaining cell division activity and callus growth [109].

One can imagine a similar scenario for wound-induced callus formation. Cutting through the hypocotyl triggers a cytokinin response to regenerate the removed shoot meristem. However, if the cut is too far from the apex, the developmental signals that are required for SAM organization are missing, and the establishment of the appropriate developmental domains, including the meristematic one, fails. Following a few rounds of wound-triggered cell divisions, the cells differentiate into parenchymatic cells to form the non-dividing callus tissue closing the wound. In contrast, if the basal part of the hypocotyl is cut through, the basipetal auxin transport from the shoot tip results in an auxin maximum at the basal part of the hypocotyl that is sufficient to evoke AR development (with or without previous callus formation). This might be ascribed to the presence of auxin-responsive pericycle-like cells that can initiate a normal root developmental program in response to endogenous auxin maxima [195,196]. The SAM can be replaced by axillary meristems developing from meristematic cells at the leaf axils during post-embryogenic development, and these cells are obviously not present in hypocotyls [197]. Nevertheless, emerging LRPs can be induced by cytokinin to form shoots instead of roots [198], indicating that the development of organ primordia is not determined at the early stage but is dependent on hormonal regulation.

If callus formation is a deviation from normal or regenerative developmental pathways, then the initial steps of callus initiation necessarily converge on those pathways [48,66], which, however, gradually fades away in the absence of the strictly controlled feedback regulations ensuring tissue/organ development. It is, therefore, not easy to decide at what step we can talk about a “callus” instead of an ill-developing organ primordium. This is why the many attempts to investigate the transcriptional pattern before or at the time of the first cell division in response to callus induction, e.g., [87,88], could not give a clear view of the nature of the callus tissue itself. An alternative approach is to analyze gene expression in established in vitro callus cultures. However, in vitro, cultured cells still express a high number of genes, and the identification of key regulators is not an easy task. Surprisingly, comparing several transcriptional datasets obtained from callus cells at various phases of in vitro culture highlighted only a handful of genes (Table 4). Interestingly, these genes include *ARF5/MP*, *AIL6/PLT3*, *SPT*, and *WRKY23*, which code for transcription factors and are expressed embryonically as well as postembryonically in those developmental domains which contribute to meristem formation, maintenance, and organ primordium formation. It was demonstrated that the ARF5/MP, AIL6/PLT3, and WRKY23 factors could positively control both the maintenance of the stem cell fate in meristems and tissue differentiation in organ primordia in a dose-dependent manner [103,104,105,111,120,138].

The gene expression domain of these factors during the early phases of embryogenesis and organ formation is the internal domains of the embryo and the organ primordia comprising the dividing ground tissue with pre-vascular cells. Shoot and root meristem initials, as well as pro-cambial cells, arise within this domain due to the establishment of spatially controlled hormonal and molecular feedback mechanisms. During callus formation, however, these feedback mechanisms are not formed due to disturbed developmental control (see earlier). Therefore, callus cells are blocked in a kind of pre-meristematic state and keep the associated gene expression signature, including the expression of the *ARF5/MP*, *AIL6/PLT3*, *SPT*, *WRKY23* and the further 17 genes I discussed above (Table 4). The function of some of these genes is maintaining the cell division activity until appropriate hormonal signals are provided exogenously or endogenously. This pre-meristematic tissue state might explain the organogenetic potential of most callus tissues. Under appropriate conditions, endogenous hormone gradients can reestablish on the surface of the cell colonies to organize shoot- or root meristem or both (i.e., somatic embryo), a phenomenon widely used in plant biotechnology [19]. Obviously, as callus growth, metabolic and hormonal gradients may establish within the tissue leading to various degrees of cellular heterogeneity, especially between the outer and inner domains, that can lead to the loss of cell division and/or organogenic potentials.

## 6. Conclusions

Plants as sessile organisms have developmentally and environmentally controlled continuous organ formation to ensure survival. The plant body is composed of two main organ systems: the root and the shoot, both of which are formed post-embryonically due to the function of apical meristems. That the right organ forms in the right place is ensured by short- and long-term developmental signals controlling the fates of meristem-derived cells. Plants can regenerate lost tissues and organs due to competent cells responding to injuries with cell divisions followed by cell differentiation. This differentiation step is under the control of the short- and long-term signals of the damaged region, ensuring that the proper cell, tissue, or organ forms in the right place. However, if these signals are not present or seriously disturbed, cells may continue to divide, forming an ill-organized organ primordium. This primordium cannot establish the inner meristematic and the outer non-dividing domains, which mutually regulate each other, but get blocked in a kind of pre-meristematic ground-tissue state. This state is characterized, among other things, by the expression of the *ARF5/MP*, *AIL6/PLT3*, *SPT*, and *WRKY23* TF genes, the *DI21* gene with an unknown function, and several other genes that have been implicated in provascular/procambial cells. In the presence of auxin and cytokinin (either exogenous or endogenous), the cells further divide in an unorganized way to form what is known as in vitro callus tissue.

## Figures and Tables

**Table 1:** Gene ontology (GO) enrichment of the 400 common genes expressed in Arabidopsis aerial and root explants on the fourth day of CIM-mediated callus initiation (made at ThaleMine; https://bar.utoronto.ca/thalemine/, last accessed on 3 July 2023 [91]).

GO Term	*p*-Value	Matches
mitotic cell cycle phase transition [GO:0044772]	1.113797 × 10^−4^	12
hormone-mediated signaling pathway [GO:0009755]	1.531419 × 10^−4^	43
cell cycle phase transition [GO:0044770]	1.660908 × 10^−4^	13
auxin-activated signaling pathway [GO:0009734]	2.028745 × 10^−4^	20
cellular response to auxin stimulus [GO:0071365]	2.297677 × 10^−4^	20
response to auxin [GO:0009733]	3.177196 × 10^−4^	26
cellular response to hormone stimulus [GO:0032870]	3.547532 × 10^−4^	41
cellular response to endogenous stimulus [GO:0071495]	8.612505 × 10^−4^	41
response to hormone [GO:0009725]	0.001567	54
response to endogenous stimulus [GO:0009719]	0.002949	54
pattern specification process [GO:0007389]	0.014219	15
root system development [GO:0022622]	0.014762	26
root development [GO:0048364]	0.016967	27
cellular response to organic substance [GO:0071310]	0.022434	42
rRNA processing [GO:0006364]	0.022969	17
ribonucleoprotein complex biogenesis [GO:0022613]	0.029372	23
regionalization [GO:0003002]	0.033701	13
rRNA metabolic process [GO:0016072]	0.037409	17
ribosome biogenesis [GO:0042254]	0.048866	20

**Table 2:** Gene ontology (GO) enrichment of the 232 common genes upregulated in two-week-old Arabidopsis calli of various origins in comparison to the initial explants/seedlings. See the text for more detail (made at ThaleMine; https://bar.utoronto.ca/thalemine/, last accessed 3 July 2023 [91]).

GO Term	*p*-Value	Matches
response to stimulus [GO:0050896]	4.533972 × 10^−11^	110
response to chemical [GO:0042221]	1.392826 × 10^−9^	68
response to hypoxia [GO:0001666]	4.483907 × 10^−9^	19
cellular response to hypoxia [GO:0071456]	5.461530 × 10^−9^	18
response to stress [GO:0006950]	5.658327 × 10^−9^	79
response to decreased oxygen levels [GO:0036293]	6.189549 × 10^−9^	19
cellular response to decreased oxygen levels [GO:0036294]	6.509485 × 10^−9^	18
cellular response to oxygen levels [GO:0071453]	6.509485 × 10^−9^	18
response to oxygen levels [GO:0070482]	6.699425 × 10^−9^	19
response to abiotic stimulus [GO:0009628]	5.633967 × 10^−5^	48
response to salt [GO:1902074]	6.862141 × 10^−5^	22
response to organic substance [GO:0010033]	8.356267 × 10^−5^	50
cellular response to chemical stimulus [GO:0070887]	1.689590 × 10^−4^	39
response to acid chemical [GO:0001101]	6.834915 × 10^−4^	19
response to water [GO:0009415]	0.001198	18
response to endogenous stimulus [GO:0009719]	0.001254	38
cellular response to stress [GO:0033554]	0.001323	32
response to hormone [GO:0009725]	0.002230	37
response to water deprivation [GO:0009414]	0.003922	17
the biological process involved in interspecies interaction between organisms [GO:0044419]	0.004448	35
response to external biotic stimulus [GO:0043207]	0.010234	34
response to other organisms [GO:0051707]	0.010234	34
cellular response to stimulus [GO:0051716]	0.014815	54
response to biotic stimulus [GO:0009607]	0.015728	34
response to oxygen-containing compound [GO:1901700]	0.032401	36

**Table 3:** Gene ontology (GO) enrichment of the 144 common genes upregulated in two-week-old Arabidopsis calli as well as in the T87 cell suspension culture in comparison to the initial explants/seedlings. See the text for more details. (made at ThaleMine; https://bar.utoronto.ca/thalemine/, last accessed 3 July 2023 [91]).

GO Term	*p*-Value	Matches
response to stimulus [GO:0050896]	9.093059 × 10^−5^	66
response to stress [GO:0006950]	0.001507	47
response to chemical [GO:0042221]	0.005759	38
response to hypoxia [GO:0001666]	0.006900	10
response to decreased oxygen levels [GO:0036293]	0.008117	10
response to oxygen levels [GO:0070482]	0.008448	10
cellular response to hypoxia [GO:0071456]	0.020639	9
cellular response to decreased oxygen levels [GO:0036294]	0.022433	9
cellular response to oxygen levels [GO:0071453]	0.022433	9

**Table 4:** The list of the 21 genes upregulated in all investigated datasets from callus initiation to cell suspension cultures.

ID	Name	Abbreviation
AT1G19850	*AUXIN RESPONSE FACTOR 5/MONOPTEROS*	*ARF5/MP*
AT1G55610	*BRI1-LIKE 1*	*BRL1*
AT2G43510	*TRYPSIN INHIBITOR PROTEIN 1*	*ATTI1*
AT2G47260	*ATWRKY23*	*WRKY23*
AT3G01970	*ATWRKY45*	*WRKY45*
AT3G02210	*COBRA-LIKE PROTEIN 1 PRECURSOR*	*COBL1*
AT3G13380	*BRI1-LIKE 3*	*BRL3*
AT3G15720	*PECTIN LYASE-LIKE SUPERFAMILY PROTEIN*	*PLL1*
AT3G29810	*COBRA-LIKE PROTEIN 2/3 PRECURSOR*	*COBL2/3*
AT3G48580	*XYLOGLUCAN ENDOTRANSGLUCOSYLASE/HYDROLASE 11*	*XTH11*
AT3G62860	*MONOACYLGLYCEROL LIPASE 12*	*MAGL12*
AT4G15910	*DROUGHT-INDUCED 21*	*ATDI21*
AT4G36930	*SPATULA*	*SPT*
AT4G38580	*FARNESYLATED PROTEIN 6*	*ATF6*
AT5G06720	*PEROXIDASE 2/PEROXIDASE 53*	*PERX53*
AT5G10510	*AINTEGUMENTA-LIKE 6/PLETHORA 3*	*AIL6/PLT3*
AT5G14000	*ANAC084*	*ANAC084*
AT5G17760	BCS1-LIKE PROTEIN	*BCS1-L*
AT5G26220	*GAMMA-GLUTAMYL CYCLOTRANSFERASE 2;1*	*GGCT2;1*
AT5G49690	*UDP-GLYCOSYLTRANSFERASE SUPERFAMILY PROTEIN*	*UGT91C1*
AT5G50260	*CYSTEINE PROTEINASES SUPERFAMILY PROTEIN*	*CEP1*

## Data Availability

No new data were created or analyzed in this study. Data sharing is not applicable to this article.

## Supplementary Materials

The following supporting information can be downloaded at: https://www.mdpi.com/article/10.3390/ijms241713122/s1.
